# Biosynthesis of the Sex Pheromone Component (*E*,*Z*)-7,9-Dodecadienyl Acetate in the European Grapevine Moth, *Lobesia botrana,* Involving ∆11 Desaturation and an Elusive ∆7 Desaturase

**DOI:** 10.1007/s10886-021-01252-3

**Published:** 2021-03-29

**Authors:** Bao-Jian Ding, Yi-Han Xia, Hong-Lei Wang, Fredrik Andersson, Erik Hedenström, Jürgen Gross, Christer Löfstedt

**Affiliations:** 1 Department of Biology, Lund University, Sölvegatan 37, SE-223 62 Lund, Sweden; 2 Department of Chemical Engineering, Mid Sweden University, SE-851 70 Sundsvall, Sweden; 3 Federal Research Centre for Cultivated Plants, Institute for Plant Protection in Fruit Crops and Viticulture, Julius Kühn-Institut, Dossenheim, Germany

**Keywords:** *In vivo *labeling experiment, Pheromone gland, Transcriptome, Gene functional characterization, Acyl-CoA oxidase, Chain shortening

## Abstract

The European grapevine moth, *Lobesia botrana*, uses (*E*,*Z*)-7,9-dodecadienyl acetate as its major sex pheromone component. Through *in vivo *labeling experiments we demonstrated that the doubly unsaturated pheromone component is produced by ∆11 desaturation of tetradecanoic acid, followed by chain shortening of (*Z*)-11-tetradecenoic acid to (*Z*)-9-dodecenoic acid, and subsequently introduction of the second double bond by an unknown ∆7 desaturase, before final reduction and acetylation. By sequencing and analyzing the transcriptome of female pheromone glands of *L. botrana*, we obtained 41 candidate genes that may be involved in sex pheromone production, including the genes encoding 17 fatty acyl desaturases, 13 fatty acyl reductases, 1 fatty acid synthase, 3 acyl-CoA oxidases, 1 acetyl-CoA carboxylase, 4 fatty acid transport proteins and 2 acyl-CoA binding proteins. A functional assay of desaturase and acyl-CoA oxidase gene candidates in yeast and insect cell (Sf9) heterologous expression systems revealed that Lbo_PPTQ encodes a ∆11 desaturase producing (*Z*)-11-tetradecenoic acid from tetradecanoic acid. Further, Lbo_31670 and Lbo_49602 encode two acyl-CoA oxidases that may produce (*Z*)-9-dodecenoic acid by chain shortening (*Z*)-11-tetradecenoic acid. The gene encoding the enzyme introducing the E7 double bond into (*Z*)-9-dodecenoic acid remains elusive even though we assayed 17 candidate desaturases in the two heterologous systems.

**Supplementary Information:**

The online version contains supplementary material available at 10.1007/s10886-021-01252-3.

## Introduction

The European grapevine moth, *Lobesia botrana*, belongs to the family Tortricidae (Lepidoptera). It feeds on grapes, causing serious yield losses as well as increasing susceptibility to fungal infections (Ioriatti et al. 2011). It is among the most economically serious pests in vineyards in Europe, as well as in Chile, Argentina and California, where *L. botrana* was accidentally introduced (Gonzales 2010; Varela et al. 2010; Witzgall et al. 2010). The use of sex pheromone-based strategies for pest control is considered an environmentally safe management approach. Pheromone-mediated mating disruption of *L. botrana* is an effective technique for grape protection and is currently used on about 140,000 ha in the European wine-growing area in the European Union (Ioriatti et al. 2011).

The sex pheromone components of *L. botrana* were identified in the 1970s and 1980s. The major pheromone component is (*E*,*Z*)-7,9-dodecadienyl acetate (E7,Z9–12:OAc) (Buser et al. 1974; Roelofs et al. 1973). Later, (*E*,*Z*)-7,9-dodecadienol (E7,Z9–12:OH) and (*Z*)-9-dodecenyl acetate (Z9–12:OAc) were reported as minor pheromone components (Arn et al. 1988). More recently, (*E*)-7-dodecenyl acetate, and (*E,E*)- and (*Z,E*)-isomers of 7,9,11-dodecatrienyl acetate were identified in the pheromone gland of *L. botrana* (Witzgall et al. 2005). However, to date, the pathway for biosynthesis of sex pheromone has not been investigated, and the enzymes involved in biosynthesis are unknown. Elucidation of the mechanisms of pheromone biosynthesis in *L. botrana* is not only of fundamental interest but could also provide genes necessary for biological production of grapevine moth pheromone in cell and plant factories for the use in pheromone-based pest control (Ding et al. 2014; Hagström et al. 2013; Löfstedt and Xia 2020; Xia et al. 2020).

Compared to other organisms in which fatty acyl desaturases are largely involved in normal cellular lipid metabolism, moth desaturases have evolved to perform specialized functions in the biosynthesis of sex pheromone components. Desaturases introduce double bonds in specific positions of fatty acids, and are responsible for much of pheromone diversity among different moth species. A wide range of desaturases has been characterized in various moths species, including: Δ5 desaturases that introduce double bonds into tetradecanoic acid for production of the fatty acid pheromone precursor (*Z*)-5-tetradecenoic acid in *Ctenopseustis obliquana* and *C. herana* (Hagström et al. 2014); a Δ6 desaturase that introduces an E6 double bond into the major pheromone component of *Antheraea pernyi* (Wang et al. 2010); several Δ9 desaturases (from a range of species) that introduce double bonds into saturated or unsaturated fatty acids of C_12_-C_18_ chain length (Liu et al. 2004; Liu et al. 2002; Rodríguez et al. 2004; Rosenfield et al. 2001); a Δ10 desaturase that introduces a double bond in hexadecanoic acid to produce the pheromone precursor (*Z*)-10-hexadecenenoic acid in *Planotortrix octo* (Hao et al. 2002); several Δ11 desaturases that produce Δ11-unsaturated fatty acids (Knipple et al. 1998); two ∆11/∆12 desaturases in *Spodoptera exigua* and *S. litura* that introduce double bonds into both saturated and unsaturated fatty acids to produce (*Z*)-11-hexadecenoic acid (Z11–16:acid) and (*Z*,*E*)-9,12-tetradecadienoic acid (Xia et al. 2019); a Δ11/Δ13 multifunctional desaturase in *Thaumetopoea pityocampa* that produces Z11–16:acid, 11-hexadecynoic acid and (*Z*)-13-hexadecen-11-ynoic acid (Serra et al. 2007); Δ14 desaturases in *Ostrinia* species that introduce double bonds into palmitic acid to produce (*Z*)- and (*E*)-14-hexadecenoic acids (Roelofs et al. 2002); and a terminal desaturase in *Operophtera brumata* that introduces a double bond into the methyl terminus of the carbon chain of *Z*11,*Z*14,*Z*17-eicosatrienoic acid to produce Z11,Z14,Z17,19-eicosatetraenoic acid (Ding et al. 2011).

In addition to desaturases, moth pheromone biosynthesis involves other enzymes that contribute to structural diversity. β-Oxidases and elongases are considered to combine with desaturases to determine the basic structures of pheromone fatty acyl precursors (Löfstedt et al. 2016) but, so far, no enzymes involved in chain-shortening havebeen identified and characterized. Fatty-acyl reductases (FARs) with different substrate specificities are responsible for reducing fatty acyl moieties to alcohols, and have been functionally characterized in several moth species (Lassance et al. 2010; Löfstedt et al. 2016; Moto et al. 2003).

In the present study, we performed *in vivo *labeling experiments to investigate the pheromone biosynthetic pathway in *L. botrana.* We did high-throughput sequencing of the *L. botrana* pheromone gland transcriptome and identified candidate genes that might be involved in pheromone biosynthesis. Finally, we functionally characterized several of these candidate genes in yeast and Sf9 heterologous systems.

## Methods and Materials

### Insects

Pupae of *L. botrana* were obtained from a rearing facility at the Julius Kühn Institut (JKI), Federal Research Centre for Cultivated Plants, Institute for Plant Protection in Fruit Crops and Viticulture, Siebeldingen, Germany. Larvae were reared in 500 ml polypropylene (PP) cups (Huthamaki, Alf, Germany) on a semi-synthetic diet, according to the protocol described by Markheiser et al. (2018). Male and female pupae were kept separately in a climate chamber at 23 ± 1 °C under a 17 h:7 h Light: Dark photoperiod and 70% RH. After emergence, adults were fed with 10% honey solution, with two- to three-day-old virgin females being used for experiments throughout this study.

### Chemicals

[12,12,12-^2^H_3_] Dodecanoic acid (D_3_–12:acid), [14,14,14-^2^H_3_] tetradecanoic acid (D_3_–14:acid), and [16,16,16-^2^H_3_] hexadecanoic acid (D_3_–16:acid) were purchased from Larodan AB (Malmö, Sweden). (*Z*)-11-[13,13,14,14,14-^2^H_5_] Tetradecenoic acid (D_5_-Z11–14:acid) was synthesized as described in Zhu et al. (1996). (*Z*)-9-[12,12,12-^2^H_3_] Dodecenoic acid (D_3_-Z9–12:acid) was synthesized as described in Supplementary File 1. Other fatty acid and pheromone standards were available in our laboratory and were of various origin.

### Labeling Experiments and Sample Preparation

The deuterium-labeled potential precursor acids D_3_–16:acid, D_3_–14:acid, D_3_–12:acid, D_5_-Z11–14:acid, and D_3_-Z9–12:acid were dissolved separately in dimethylsulphoxide (DMSO) at 40 μg/μl. About 1 h into scotophase, 0.4 μl of a solution of a potential precursor was applied topically to the pheromone gland of females in a group. The same volume of DMSO was applied as a control to females in another group. After 1 h incubation, pheromone glands were excised and five glands pooled in a 250 μl insert (in a 1.5 ml glass vial) to which 20 μl *n*-heptane was added. After extracting glands for 30 min at room temperature, the solvent, which contained pheromone components, was transferred into a new vial. The remaining lipids in the residue were subsequently extracted with 100 μl chloroform/methanol (2:1 v:v) at room temperature overnight. After extraction, base methanolysis was conducted as described in Bjostad and Roelofs (1984).

### Gas Chromatography/Mass Spectrometry (GC/MS)

Pheromone gland extracts and methylated fatty acyl compounds were analyzed using a Hewlett-Packard (HP) 5975 mass selective detector coupled to an HP 6890 series gas chromatograph, equipped with a polar column (HP-INNOWax, 30 m × 0.25 mm, 0.25 μm film thickness) or an Agilent 5975C mass selective detector coupled to an Agilent 7890A series gas chromatograph equipped with a non-polar column (HP-5MS, 30 m × 0.25 mm, 0.25 μm film thickness). Helium was used as carrier gas (average velocity: 33 cm sec^−1^). The injector was configured in splitless mode at 250 °C.

For analysis of pheromone gland extracts, the column oven temperature was set at 80 °C for 1 min, then increased to 190 °C at 10 °C min^−1^, held for 10 min, and finally increased to 230 °C at 4 °C min^−1^, then held for 10 min. Incorporation of deuterium label into the pheromone components was detected by selected ion monitoring (SIM) mode (Table 1).

**Table 1:** Selected ion monitoring (SIM) mode used for detecting incorporation of deuterium label into the pheromone components

Compound	SIM (*m/z*)	Compound	SIM (*m/z*)	Compound	SIM (*m/z*)
Z9–12:OAc^1^	166, 61	E7,Z9–12:OAc	164, 224, 61	E7,Z9–12:OH	164, 182, 31
D_3_-Z9–12:OAc	169, 61	D_3_-E7,Z9–12:OAc	167, 227, 61	D_3_-E7,Z9–12:OH	167, 185, 31
D_5_-Z9–12:OAc	171, 61	D_5_-E7,Z9–12:OAc	169, 229, 61	D_5_-E7,Z9–12:OH	169, 187, 31

^1^Compound acronyms refer to geometry across double bond, position of unsaturation, carbon chain length, functionality and total number of D labels; e.g., D_5_-Z9–12:OAc = (*Z*)-9-dodecenyl acetate with five D atoms

For fatty acid methyl esters (FAMEs), the oven temperature was set at 80 °C for 1 min, then increased to 230 °C at 10 °C min^−1^ and held for 10 min. Incorporation of deuterium label into pheromone precursors was detected in the SIM mode, as described in Table 2. Full scans, from *m/z* 30–400, were for mass spectra. Compounds were identified by comparison of retention times and mass spectra with corresponding standards.

**Table 2:** Selected ion monitoring (SIM) mode used for detecting incorporation of deuterium label into the fatty acyl methyl ester (FAME) pheromone precursors

Saturated FAME	SIM (*m/z*)	Mono-unsaturated FAME	SIM (*m/z*)	Di-unsaturated FAME	SIM (*m/z*)
16:Me^1^	270	Z11-14:Me	166, 240	E7,Z9–12:Me	136, 210
D_3_–16:Me	273	D_3_-Z11–14:Me	169, 243	D_3_-E7,Z9–12:Me	139, 213
14:Me	242	D_5_-Z11–14:Me	171, 245	D_5_-E7,Z9–12:Me	141, 215
D_3_–14:Me	245	Z9–12:Me	138, 212	–	–
12:Me	214	D_3_-Z9–12:Me	141, 215	–	–
D_3_–12:Me	217	D_5_-Z9–12:Me	143, 217	–	–

^1^FAME acronyms refer to geometry across double bonds, position of unsaturation, carbon chain length, esterification and total number of D labels; e.g., D_5_-E7,Z9–12:Me refers to methyl (*E*,*Z*)-7,9-dodecadienoate with five D atoms

### RNA Isolation, cDNA Library Construction and Illumina Sequencing

Approximately 50 pheromone glands of two- to three-day-old virgin females of *L. botrana* were collected for transcriptome sequencing. Total RNA of the glands was extracted using the TRIzol® reagent (Invitrogen). As control tissue, 25 abdominal tips from males were collected and treated the same way. We used Agilent 2100 Bioanalyzer system to check the RNA integrity and quantitation. Total RNA was sent to Novogene (Hong Kong) for Illumina sequencing.

### De Novo Assembly and Bioinformatics Analysis

Transcriptome assembly was accomplished using Trinity (Grabherr et al. 2011) to assemble the clean reads de novo. Gene function was annotated based on the databases of Nr (NCBI non-redundant protein sequences), Nt (NCBI non-redundant nucleotide sequences), Pfam (Protein family), KOG/COG (Clusters of Orthologous Groups of proteins), Swiss-Prot (a manually annotated and reviewed protein sequence database), KO (KEGG Ortholog database) and GO (Gene Ontology).

### Quantification of Gene Expression Level

Gene expression levels were estimated by RSEM (Li and Dewey 2011) for each sample: clean data were mapped back onto the assembled transcriptome and read count for each gene was obtained from the mapping results. Gene Ontology (GO) enrichment analysis of the differentially expressed genes (DEGs) was implemented by the GOseq R packages based on Wallenius non-central hyper-geometric distribution (Young et al. 2010), which can adjust for gene length bias in DEGs. They were converted into values of FPKM (expected number of Fragments Per Kilobase of transcript sequence per Millions base pairs-sequenced in RNA-seq). FPKM is the most common method of estimating gene expression levels, which considers the effects of both sequencing depth and gene length on counting of fragments (Van Verk et al. 2013).

### Phylogenetic Analysis

Local BLAST was performed using Geneious software. Desaturase sequences used for phylogenetic reconstructions were retrieved from the GenBank (http://www.ncbi.nlm.nih.gov) database. MAFFT alignment (Katoh and Standley 2013; Katoh et al. 2002) with scoring matrix Blosum62 was performed in Geneious. Phylogenetic analysis was performed in IQtree 2.0-rc2 (http://www.iqtree.org; last accessed: Nov-11, 2020) using ultrafast bootstrap (Hoang et al. 2018; Minh et al. 2020) with 5000 replicates. We used Geneious (version 9.1, created by Biomatters, available from http://www.geneious.com/) to visualize and annotate the phylogenetic tree. The terminology for desaturases introduced by Knipple et al. (2002), based on the most prevalent signature motif within a supported grouping of lepidopteran sequences, was used when appropriate.

### Functional Assay in Yeast

For the construction of a yeast expression vector containing a candidate desaturase gene, specific primers with attB1 and attB2 sites incorporated were designed for amplifying the ORF. The PCR products were subjected to agarose gel electrophoresis and purified using the Wizard® SV Gel and PCR Clean up system (Promega). The ORF was cloned into the pDONR221 vector in the presence of BP clonase (Invitrogen). After confirmation by sequencing, the correct entry clones were selected and subcloned to pYEX-CHT vector (Patel et al. 2003), and recombinant constructs analyzed by sequencing. The pYEX-CHT recombinant expression vectors harboring the *L. botrana* desaturase genes were introduced into the double deficient *ole1*/*elo1* strain (MATa elo1::HIS3 ole1::LEU2 ade2 his3 leu2 ura3) of the yeast *Saccharomyces cerevisiae*, defective in both desaturase and elongase functions (Schneiter et al. 2000), using the S.c. easy yeast transformation kit (Life technologies). For selection of uracil and leucine prototrophs, the transformed yeast was allowed to grow on SC plate containing 0.7% YNB (w/o aa, with ammonium sulfate), a complete drop-out medium lacking uracil and leucine (Formedium), 2% glucose, 1% tergitol (type Nonidet NP-40, Sigma), 0.01% adenine (Sigma) and 0.5 mM oleic acid (Sigma) as an extra fatty acid source. After 4 days at 30 °C, individual colonies were picked up to inoculate 10 ml selective medium, which was grown at 30 °C at 300 rpm for 48 h. Yeast cultures were diluted to an OD600 of 0.4 in 10 ml fresh selective medium containing 2 mM CuSO_4_ with supplementation of a biosynthetic precursor. Each FAME precursor (14:Me, E9–14:Me, Z11–14:Me, Z9–12:Me) was prepared at a concentration of 100 mM in 96% ethanol and added to reach a final concentration of 0.5 mM in the culture medium (Ding and Löfstedt 2015; Ding et al. 2011). We used FAMEs as supplemented precursors (here and in the assay with insect cell lines below) because they are more soluble in the medium than free fatty acids. Yeasts were cultured at 30 °C with Cu^2+^-induction. After 48 h, yeast cells were harvested by centrifugation at 3000 rpm and the medium was discarded. The pellets were stored at −80 °C until fatty acid analysis.

### Functional Assay in Sf9 Cells

The expression construct for Lbo_ACOs in the BEVS donor vector pDEST8 was made by LR reaction. Recombinant bacmids were made according to instructions for the Bac-to-Bac™ system given by the manufacturer Invitrogen using DH10MEmBacY (Geneva Biotech). Baculovirus generation was done using Sf9 cells (Invitrogen), Ex-Cell 420 medium (Sigma) and baculoFECTIN II (OET). Virus was then amplified once to generate a P2 virus stock using Sf9 cells and Ex-Cell 420 medium. The virus titer in the P2 stock was determined using the BaculoQUANT all-in-one qPCR kit (OET). Insect cells lines Sf9 were diluted to 2 × 10^6^ cells/ml. Expression was done in 20 ml cultures in Ex-Cell 420 media, at virus additions (MOI 1). The cultures were incubated in 125 ml Erlenmeyer flasks (100 rpm, 27 °C), with Z11–14:Me supplemented at a concentration of 0.25 mM on the second day. On the fourth day of expression, 7.5 ml samples were taken from the culture and centrifuged for 15 min at 4500 x g at 4 °C. The pellets were stored at −80 °C until fatty acid analysis. Aliquots were also taken for visualization in the fluorescence microscope of YFP expression from the virus backbone.

### Fatty Acid Analysis of Yeast and Sf9 Cells

Total lipids were extracted using 3.75 ml of methanol/chloroform (2:1, *v*/v) in a glass tube. One ml of HAc (0.15 M) and 1.25 ml of water were added to the tube to wash the chloroform phase. Tubes were vortexed vigorously and centrifuged at 2000 rpm for 2 min. The bottom chloroform phase (ca. 1 ml), containing the total lipids, was transferred to a new glass tube. FAMEs were made from this total lipid extract. The solvent was evaporated to dryness under gentle nitrogen flow. One milliliter of sulfuric acid, 2% in methanol, was added to the tube, vortexed vigorously, and incubated at 90 °C for 1 h. After incubation, 1 ml of water was added, mixed well, and then 1 ml of *n*-hexane used to extract FAMEs. FAME samples were subjected to GC/MS analysis on an Agilent 8890 GC/Agilent 7693MS.

## Results

### Biosynthetic Pathway

**Fig. 1:**
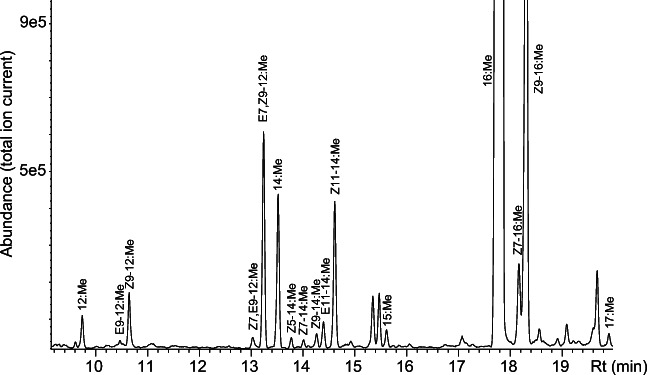
**Fatty acid profile of** ***Lobesia botrana*** **female pheromone glands.** Mass chromatograms of fatty acids in the *Lobesia botrana* pheromone gland in the form of fatty acid methyl esters. Acronyms refer to geometry across double bonds, position of unsaturation, carbon chain length and esterification; e.g., E9–12:Me refers to methyl (*E*)-9-dodecenoate

Fatty acyl moieties identified from gland extracts are shown in Fig. 1. Relatively high amounts of monounsaturated Z11–14:acid, Z9–12:acid and doubly unsaturated E7,Z9–12:acid were found, together with small amounts of several other unsaturated fatty acids, including (*E*)-9-dodecenoic acid (E9–12:acid), (*Z*)-5-tetradecenoic acid (Z5–14:acid), (*Z*)-7-tetradecenoic acid (Z7–14:acid), (*Z*)-9-tetradecenoic acid (Z9–14:acid), (*E*)-11-tetradecenoic acid (E11–14:acid), (*Z*)-7-hexadecenoic acid (Z7–16:acid) and (*Z*,*E*)-7,9-dodecadienoic acid (Z7,E9–12:acid). No doubly unsaturated C_14_ or C_16_ fatty acids were detected (Fig. 1).

**Fig. 2:**
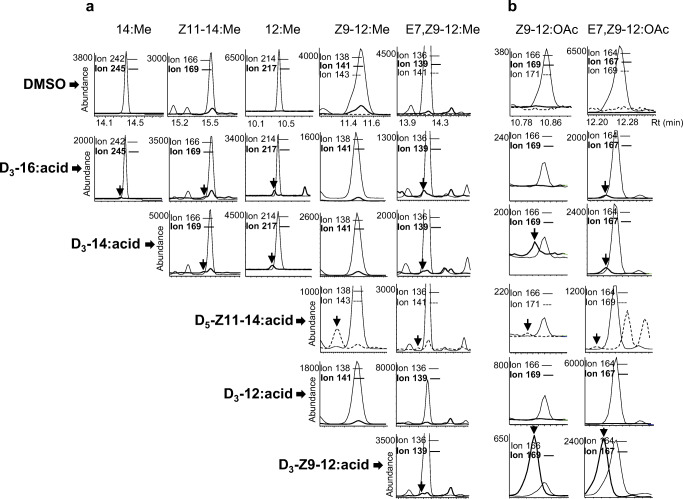
**Incorporation of deuterium labels into fatty acyl precursors and pheromone components in** ***Lobesia botrana.*** Representative chromatograms obtained on INNOWax column. Fatty acyl precursors were analyzed as corresponding methyl esters. Label incorporation from [16,16,16-^2^H_3_] hexadecanoic acid (D_3_–16:acid), [14,14,14-^2^H_3_] tetradecanoic acid (D_3_–14:acid), (*Z*)-11-[13,13,14,14,14-^2^H_5_] tetradecenoic acid (D_5_-Z11–14:acid), [12,12,12-^2^H_3_] dodecanoic acid (D_3_–12:acid) and (*Z*)-9-[12,12,12-^2^H_3_] dodecenoic acid (D_3_-Z9–12:acid) were indicated by arrows in comparison with that from a DMSO solvent control. a. Ions at *m/z* 242 and 214 were used to monitor the native methyl tetradecanoate (14:Me) and dodecanoate (12:Me), respectively. Ions at *m/z* 245 and 217 monitored three deuterium atoms incorporated into 14:Me and 12:Me, respectively. Ions at *m/z* 166, 138, 136 monitored native methyl (*Z*)-11-tetradecenoate (Z11–14:Me), methyl (*Z*)-9-dodecenoate (Z9–12:Me), methyl (*E*,Z)-7,9-dodecadienoate (E7,Z9–12:Me), respectively. Ions at *m/z* 169, 141 and 139 monitored three deuterium atoms incorporated into Z11–14:Me, Z9–12:Me and E7,Z9–12:Me, respectively. Ions at *m/z* 143 and 141 monitored five deuterium atoms incorporated into Z9–12:Me and E7,Z9–12:Me, respectively. b. Ions at *m/z* 166 and 164 monitored native (*Z*)-9-dodecenyl acetate (Z9–12:OAc) and (*E*,*Z*)-7,9-dodecadienyl acetate (E7,Z9–12:OAc), respectively. Ions at *m/z* 169 and 167 monitored three deuterium atoms incorporated into Z9–12:OAc and E7,Z9–12:OAc. Ions at *m/z* 171 and 169 monitored five deuterium atoms incorporated into Z9–12:OAc and E7,Z9–12:OAc. The deuterium-labeled compounds elute earlier than unlabeled compounds because of isotope effects (Matucha et al. 1991)

**Fig. 3:**
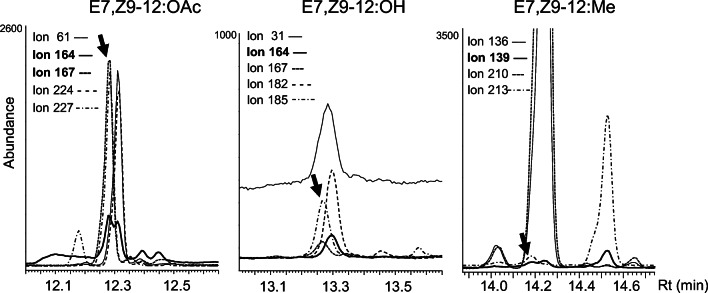
**Incorporation of deuterium label from (*Z*)-9-[12,12,12-**^**2**^**H**_**3**_**] dodecenoic acid (D**_**3**_**-Z9–12:acid) into pheromone components in** ***Lobesia botrana*** **proved Δ7 desaturation on Z9–12:Acid.** Representative chromatograms obtained from analysis on INNOWax column. Ions at *m/z* 164 and 224 monitored native (*E*,*Z*)-7,9-dodecadienyl acetate (E7,Z9–12:OAc). Ions at *m/z* 167 and 227 monitored three deuterium atoms incorporated into E7,Z9–12:OAc. Ions at *m/z* 164 and 182 monitored native (*E*,*Z*)-7,9-dodecadienol (E7,Z9–12:OH). Ions at *m/z* 167 and 185 monitored three deuterium atoms incorporated into E7,Z9–12:OH. In addition, reference ions at *m/z* 31 and 61 monitored the related alcohol and acetate, respectively (Friedel et al. 1956; Sharkey et al. 1959). Ions at *m/z* 136 and 210 monitored native methyl (E,Z)-7,9-dodecadienoate (E7,Z9–12:Me). Ions at *m/z* 139 and 213 monitored three deuterium atoms incorporated into E7,Z9–12:Me. The deuterium-labeled compounds indidated by arrows elute earlier than the unlabeled compounds because of isotope effects (Matucha et al. 1991)

Label from D_3_–16:acid was incorporated into 14:acid, 12:acid, Z11–14:acid, and E7,Z9–12:acid (Fig. 2a), as well as the major pheromone compound E7,Z9–12:OAc (Fig. 2b). Similarly, label from D_3_–14:acid was incorporated into 12:acid, Z11–14:acid, E7,Z9–12:acid (Fig. 2a), and the pheromone compounds E7,Z9–12:OAc and Z9–12:OAc (Fig. 2b). Label from D_5_-Z11–14:acid was incorporated into Z9–12:acid and E7,Z9–12:acid (Fig. 2a), and the corresponding acetates (Fig. 2b). Label from D_3_-Z9–12:acid was incorporated into E7,Z9–12:acid (Fig. 2a), E7,Z9–12:OAc and Z9–12:OAc (Fig. 2b), as well as the corresponding alcohol E7,Z9–12:OH (Fig. 3). Label incorporation into acyl precursors was generally low in this study, except from D_5_-Z11–14:acid into (chain-shortened) D_5_-Z9–12:acid (Fig. 2a). Compared to other labeling results, the incorporation rates from D_3_-Z9–12:acid into the doubly unsaturated acetate and alcohol were remarkably high (Fig. 3). However, when D_3_–12:acid was applied, no incorporation of label was detected in any of the abovementioned compounds (Fig. 2).

### Transcriptome Assembly

A total of more than 78 million raw reads were generated by Illumina HiSeq™ 2500 from the pheromone glands of *L. botrana*, resulting in about 75 million clean reads after clustering and redundancy filtering of the raw reads. Data were deposited in NCBI database under accession code PRJNA663283. The clean reads were assembled into 75,207 unigenes with a mean length of 1247 bp and the N50 length of 2061 bp (Table 3). BUSCO completeness for the assembled transcripts was 96% (Simao et al. 2015).

**Table 3:** Distribution of Unigenes size in *Lobesia botrana* female glands transcriptome assembly

Transcript length interval	200–500 bp	500-1kbp	1 k-2kbp	>2kbp	Total
Number of transcripts	25,735	19,473	16,075	13,940	75,223

### Gene Ontology (GO) Annotation

**Fig. 4:**
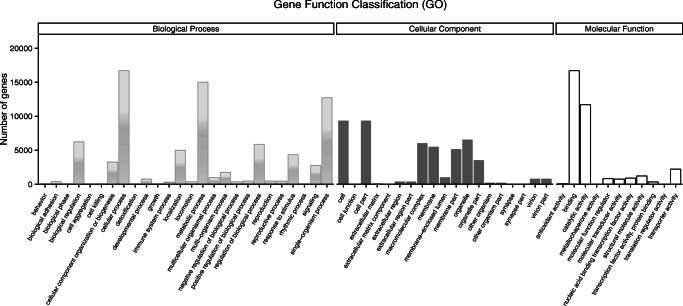
**Gene ontology (GO) classification of the** ***Lobesia botrana*** **female pheromone glands transcripts with Blast2GO program.** One unigene could be annotated into more than one GO term

The 75,207 unigenes were classified into different functional groups using BLAST2GO for Gene Ontology (GO) annotation. Based on sequence homology, 29,065 unigenes (38.64%) could be annotated. After GO annotation, the successfully annotated genes were grouped into three main GO domains: Biological Process (BP), Cellular Component (CC), Molecular Function (MF). One unigene could be annotated into more than one GO term. Each unigene was grouped into one or more GO domains (Fig. 4).

### Sex Pheromone Biosynthesis Gene Candidates

By homology search, 41 genes putatively related to sex pheromone biosynthesis were obtained, including candidate genes encoding 17 desaturases, 13 FARs, 1 fatty acid synthase (FAS), 3 acyl-CoA oxidases (ACOs), 1 acetyl-CoA carboxylase (ACC), 4 fatty acid transport proteins (FATPs) and 2 acyl-CoA binding proteins (ACBPs) (Table 4).

**Table 4:** Transcripts identified as putative pheromone biosynthesis genes in the pheromone gland of *Lobesia botrana*

	**Gene**	**Length**	**Best Blastx Match**				
**Name**	**ID**	**(aa)**	**Name**	**Acc. number**	**Species**	**E value**	**Identity (%)**
***Desaturase (DES)***							
**Lbo_NPVE_(A)**	Cluster-6761.35600	350	delta 9 desaturase	AIM40222.1	[*Cydia pomonella*]	0.0	87
**Lbo_GATD_(A)**	Cluster-6761.9745	384	desaturase	AIM40222.1	[*Cydia pomonella*]	0.0	87
**Lbo_PPTQ_(A)**	Cluster-6761.26702	337	acyl-CoA delta (11) desaturase like	AER29851.1	[*Ctenopseustis herana*]	1.00E-169	69
**Lbo_TPSQ_(A)**	Cluster-6761.53556	346	acyl-CoA delta (11) desaturase like	XP_013195132.1	[*Amyelois transitella*]	0.0	95
**Lbo_LPGQ_(A)**	Cluster-6761.38020	350	desaturase	AIM40218.1	[*Cydia pomonella*]	2.00E-179	84
**Lbo_KPSE_(A)**	Cluster-6761.53650_g1_i1	374	desaturase	AIM40223.1	[*Cydia pomonella*]	0.0	86
**Lbo_KSTE_(B)**	Cluster-6761.50373	352	delta 9 desaturase	AIM40221.1	[*Cydia pomonella*]	0.0	92
**Lbo_SATQ_(B)**	Cluster-6761.3834	345	terminal desaturase	AER29851.1	[*Ctenopseustis herana*]	0.0	70
**Lbo_SPTQ_(B)**	Cluster-6761.40238	360	acyl-CoA delta (11) desaturase like	XP_013195132.1	[*Amyelois transitella*]	0.0	96
**Lbo_RAVE1_(B)**	Cluster-6761.25528	330	fatty acyl desaturase	AHW98359.1	[*Grapholita molesta*]	0.0	92
**Lbo_RAVE2_(B)**	Cluster-6761.28035	305	fatty acyl desaturase	AHW98358.1	[*Grapholita molesta*]	1.00E-180	72
**Lbo_44,979_(B)**	Cluster-6761.44979	337	desaturase	AIM40218.1	[*Cydia pomonella*]	0.0	84
**Lbo_21731_(B)**	Cluster-6761.21731	448	desaturase	AIM40226.1	[*Cydia pomonella*]	0.0	73
**Lbo_44136_(B)**	Cluster-6761.44136	451	desaturase	AIM40226.1	[*Cydia pomonella*]	0.0	77
**Lbo_36936_(B)**	Cluster-6761.36936	447	desaturase	AIM40226.1	[*Cydia pomonella*]	0.0	66
**Lbo_28799_(B)**	Cluster-6761.28799	462	cytochrome b5-related protein	XP_004924008.1	[*Bombyx mori*]	0.0	65
**Lbo_29833_(B)**	Cluster-6761.29833	459	cytochrome b5-related protein-like	XP_028158528.1	[*Ostrinia furnacalis*]	0.0	65
***Fatty-acyl reductase (FAR)***							
**Lbo_FAR_11462**	Cluster-11,426.0	508	putative fatty acyl-CoA reductase CG5065	KPI96398.1	[*Papilio xuthus*]	0.0	69
**Lbo_FAR_11487**	Cluster-6761.11487	519	putative fatty-acyl CoA reductase CG5065	XP_014366322.1	[*Papilio machaon*]	0.0	68
**Lbo_FAR_17149**	Cluster-6761.17149	600	fatty-acyl reductase 5	ATJ44463.1	[*Helicoverpa armigera*]	0.0	74
**Lbo_FAR_22678**	Cluster-6761.22678	530	putative fatty-acyl CoA reductase 5	ALJ30239.1	[*Spodoptera litura*]	2.00E-171	47
**Lbo_FAR_22999**	Cluster-6761.22999	546	fatty-acyl CoA reductase 2	ADI82775.1	[*Ostrinia nubilalis*]	0.0	76
**Lbo_FAR_30064**	Cluster-6761.30064	510	putative fatty acyl-CoA reductase CG8306	XP_022824194.1	[*Spodoptera litura*]	0.0	73
**Lbo_FAR_33934**	Cluster-6761.33934	523	fatty-acyl CoA reductase 4	AKD01782.1	[*Helicoverpa assulta*]	0.0	64
**Lbo_FAR_34479**	Cluster-6761.34479	515	putative fatty-acyl CoA reductase CG5065 isoform X1	XP_011556963.1	[*Plutella xylostella*]	0.0	73
**Lbo_FAR_37174**	Cluster-6761.37174	516	putative fatty acyl-CoA reductase CG5065	XP_012549537	[*Bombyx mori*]	0.0	79
**Lbo_FAR_45109**	Cluster-6761.45109	528	fatty acyl-CoA reductase wat-like	XP_022823965.1	[*Spodoptera litura*]	0–0	62
**Lbo_FAR_47677**	Cluster-6761.47677	522	unnamed protein product	VVD01911.1	[*Leptidea sinapis*]	0.0	77
**Lbo_FAR_47678**	Cluster-6761.47678	536	fatty acyl-CoA reductase 1	XP_014371693.1	[*Papilio machaon*]	0.0	76
**Lbo_FAR_48939**	Cluster-6761.48939	469	hypothetical protein B5V51_2931	PCG70467.1	[*Heliothis virescens*]	0.0	69
**Lbo_FAR_50390**	Cluster-6761.50390	497	fatty-acyl CoA reductase wat-like	XP_022823965.1	[*Spodoptera litura*]	0.0	62
***Fatty-acyl synthase (FAS)***							
**FAS1**	Cluster-6761.27205	2397	fatty acid synthase 2	AKD01761.1	[*Helicoverpa assulta*]	0.0	76
***Acyl-CoA oxidase (ACO)***							
**Lbo_ACO_31670**	Cluster-6761.31670	670	putative peroxisomal acyl-coenzyme A oxidase 1	KPJ00251.1	[*Papilio xuthus*]	0.0	74
**Lbo_ACO_49554**	Cluster-6761.49554	697	probable peroxisomal acyl-coenzyme A oxidase 1	XP_022821502.1	[*Spodoptera litura*]	0.0	84
**Lbo_ACO_49602**	Cluster-6761.49602	687	peroxisomal acyl-CoA oxidase 3	AID66678.1	[*Agrotis segetum*]	0.0	72
***Acetyl-CoA Carboxylase (ACC)***							
**ACC1**	Cluster-6761.34968	2284	acetyl-CoA carboxylase	ALS92678.1	[*Helicoverpa armigera*]	0.0	89
***Fatty acid transport protein (FATP)***							
**FATP1**	Cluster-6761.26169	700	hypothetical protein B5V51_5556	PCG80606.1	[*Heliothis virescens*]	0.0	78
**FATP2**	Cluster-6761.38820	662	FATP	ACT22576.1	[*Manduca sexta*]	0.0	81
**FATP3**	Cluster-6761.42541	646	long-chain fatty acid transport protein 4-like	XP_011558256.1	[*Plutella xylostella*]	0.0	81
**FATP4**	Cluster-6761.27360	512	long-chain fatty acid transport protein 4-like	XP_011558257.1	[*Plutella xylostella*]	0.0	78
***Acyl-CoA binding protein (ACBP)***							
**ACBP1**	Cluster-6761.16126	533	acyl-CoA-binding domain-containing protein 5 isoform X1	XP_013185892.1	[*Amyelois transitella*]	8.00E-101	90
**ACBP2**	Cluster-6761.51862	508	acyl-CoA-binding domain-containing protein 5 isoform X2	XP_013185900.1	[*Amyelois transitella*]	7.00E-102	89

### Phylogenetic Analysis

**Fig. 5:**
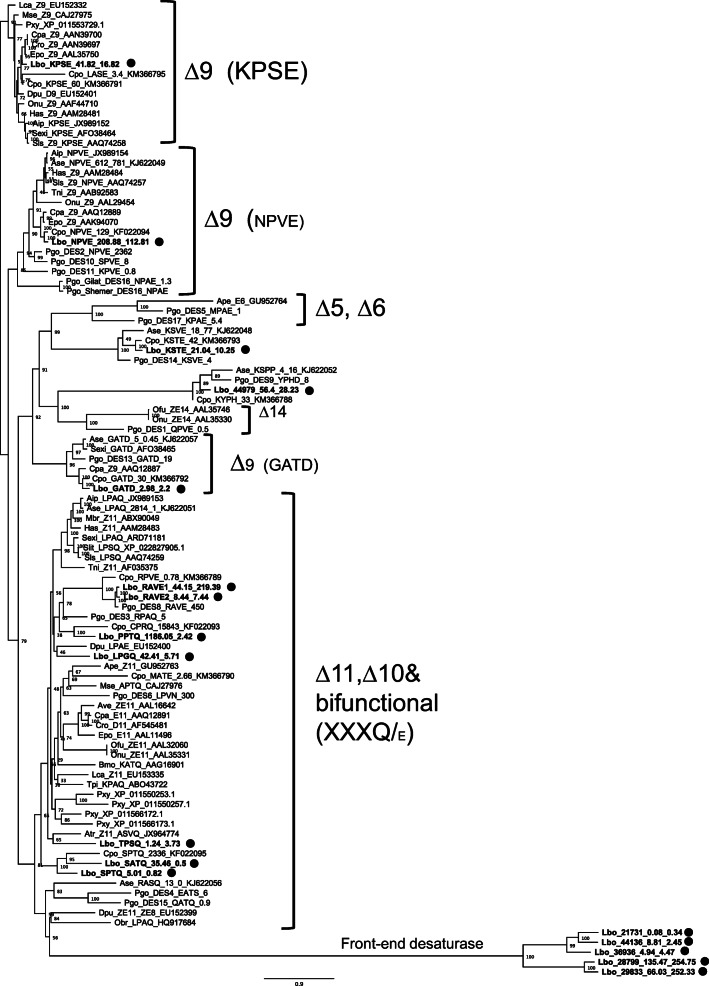
**Phylogenetic tree of desaturases identified from** ***Lobesia botrana*** **and other Lepidoptera species.** The maximum likelihood tree of selected desaturase genes constructed using amino-acid sequences. The *L. botrana* desaturases are indicated by solid dot, with expression level indicated as FPKM_PG (pheromone gland)_FPKM_MAT (male abdominal tip)

By searching the transcriptome data using desaturase His 1, 3 family motifs and the cytochrome b5 domain (Marquardt et al. 2000; Napier et al. 1999), we found 17 full-length desaturase-like genes. We next performed phylogenetic reconstructions with the 17 desaturase-like genes identified in *L. botrana*. Five genes fall into the front-end/cytochrome-b5-related clade (Fig. S2). These were subsequently treated separately due to their low similarity with first desaturases. Our analyses indicate that three of the *L. botrana* first desaturase candidates cluster into the ∆9 desaturases clade, seven fall into the clade of ∆11, ∆10 and bifunctional desaturases, and the last two did not cluster into any functionally-characterized desaturase clade (Fig. 5).

### Functional Assay of Desaturase Candidates

**Fig. 6:**
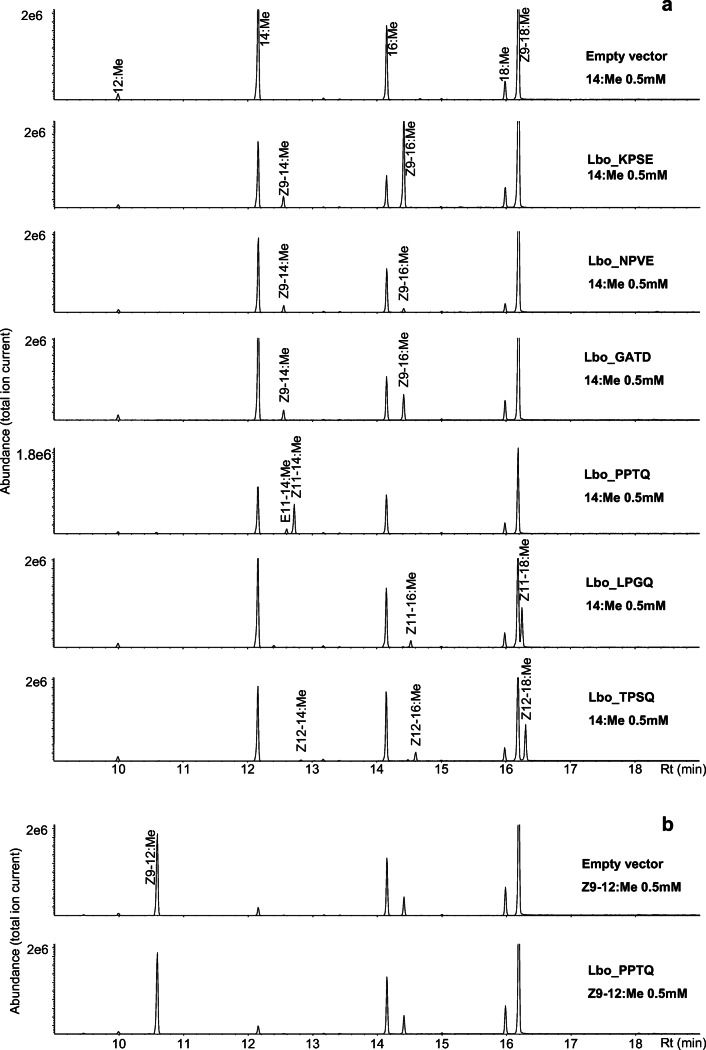
**Functional assay of the desaturase candidates in the yeast expression system.** Yeast cells carrying individual desaturase gene candidates were cultivated and fed with a) methyl tetradecanoate (14:Me) and b) methyl (*Z*)-9-dodecenoate (Z9–12:Me). The chromatogram in b from Lbo_PPTQ is representative of all the desaturases from Group A and Group B

**Fig. 7:**
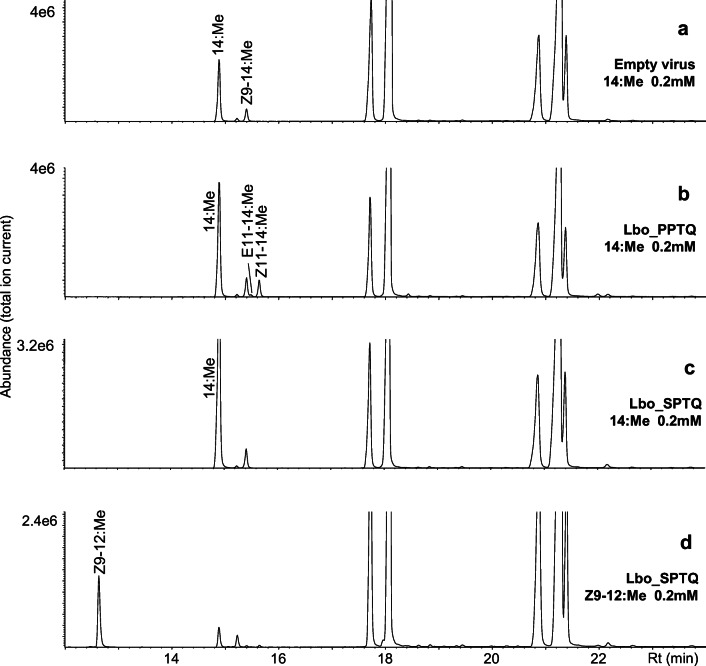
**Sf9 expression of “group B” desaturases.** a) Empty virus as negative control and b) Lbo_PPTQ as positive control. c) Desaturases from group B (Lbo_KSTE, Lbo_SATQ, Lbo_SPTQ, Lbo_RAVE1, Lbo_RAVE2, Lbo_44979, Lbo_21731, Lbo_44136, Lbo_36936, Lbo_28799, Lbo_29833) were fed with 14:Me, d) Desaturases from group B were fed with methyl (*Z*)-9-dodecenoate (Z9–12:Me). The chromatograms in c and d are from Lbo_SPTQ and they are representative of all the desaturases from Group B. Acronyms of fatty acid methyl esters refer to geometry across double bonds, position of unsaturation, carbon chain length and esterification

We heterologously expressed all the desaturase candidates in our *∆ole1/elo1* yeast system. In the first round of experiments, we fed the yeast with 14:Me as substrate; the yeast naturally produces a high amount of saturated C_16_ fatty acid precursor. Lbo_KPSE, Lbo_NPVE and Lbo_GATD produced Z9–14:acid and Z9–16:acid. Lbo_PPTQ produced Z11–14:acid, Lbo_LPGQ produced Z11–16:acid and (*Z*)-11-octadecenoic acid (Z11–18:acid). Lbo_TPSQ showed ∆12 desaturation activity, producing (*Z*)-12-tetradecenoic acid (Z12–14:acid), (*Z*)-12-hexadecenoic acid (Z12–16:acid), and (*Z*)-12-octadecenoic acid (Z12–18:acid). We assigned the name “group A” for these 6 desaturases (Fig. 6a). For those not showing any activity in this round, the name “group B” was assigned. In the second round of experiments, we fed the yeast expressing each desaturase candidate with Z9–12:Me, but none of the desaturases showed any evidence of ∆7 desaturation (Fig. 6b). The chromatogram is from Lbo_PPTQ, but is representative of all the desaturases from Group A and Group B. We further supplemented group B desaturases with E9–14:Me and Z11–14:Me, but did not see any doubly unsaturated product (E9,Z11–14:Me). We also expressed all the group B desaturases in the Sf9 system (Fig. 7). Thus, in the third round of experiments, all the “group B” desaturase were fed with 14:Me to test if there were any activity at all in the Sf9 cells, since they are not active in the yeast system. Figure 7c is a chromatogram from Lbo_SPTQ fed with 14:Me, which is representative of all the candidates from “group B”. None of them showed any desaturation activity. In the fourth round of experiments, all “group B” desaturases and the Lbo_PPTQ were fed with Z9–12:Me in Sf9 cells. None of them showed ∆7 desaturation activity, neither producing any ∆7 unsaturated monoenes nor dienes. The chromatogram from Lbo_SPTQ fed with Z9–12:Me (Fig. 7d) is representative of all the candidates from “group B”, with all resulting in similar chromatograms.

### Acyl-CoA Oxidase (ACO)

**Fig. 8:**
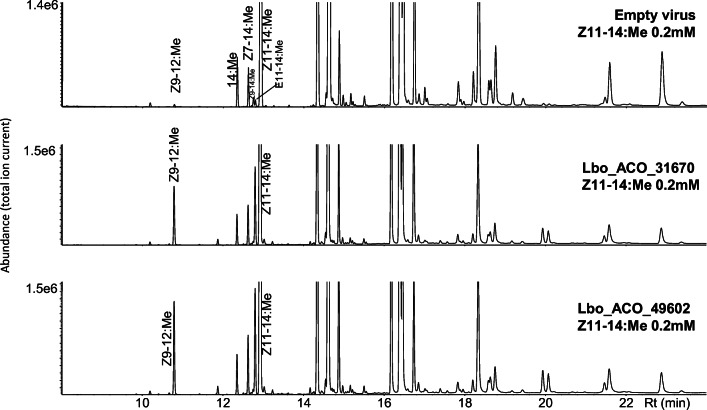
**Functional assay of the acyl-CoA oxidase candidates in the Sf9 expression system.** Sf9 cells were fed with methyl (*Z*)-11-tetradecenoate (Z11–14:Me) as substrate for the chain-shortening enzymes. Acronyms of fatty acid methyl esters refer to geometry across double bonds, position of unsaturation, carbon chain length and esterification

We found three full-length acyl-CoA oxidases (Table 4) in the *L. botrana* transcriptome and functionally expressed the two most highly expressed ones, i.e., ACO_31670 and ACO_49602 (Table 5) in the Sf9 expression system (Fig. 8). We found a significant peak of Z9–12:Me in the chromatograms of cells expressing ACO_31670 and ACO_49602 when Z11–14:Me was added, but only a tiny peak in cells harboring the empty virus (two replicates). Hypothesizing that Lbo_PPTQ introduces the first double bond in tetradecanoic acid and that the second double bond is introduced by another desaturase immediately after chain shortening of the Z11–14:acid intermediate, we also co-expressed Lbo_PPTQ, ACO_31670, ACO_49602 and all “group B” desaturases in Sf9 cells in a separate experiment. We did not find any trace of methyl E7,Z9–12:Me (data not shown).

**Table 5:** Expression levels of acyl-CoA oxidase gene candidates from *Lobesia botrana*

Acyl-CoA oxidase genes	Expression level (PG/MG) [FPKM]
Lbo_31670	178 / 28
Lbo_49602	103 / 1.5
Lbo_49554	12 / 1.7

## Discussion

**Fig. 9:**
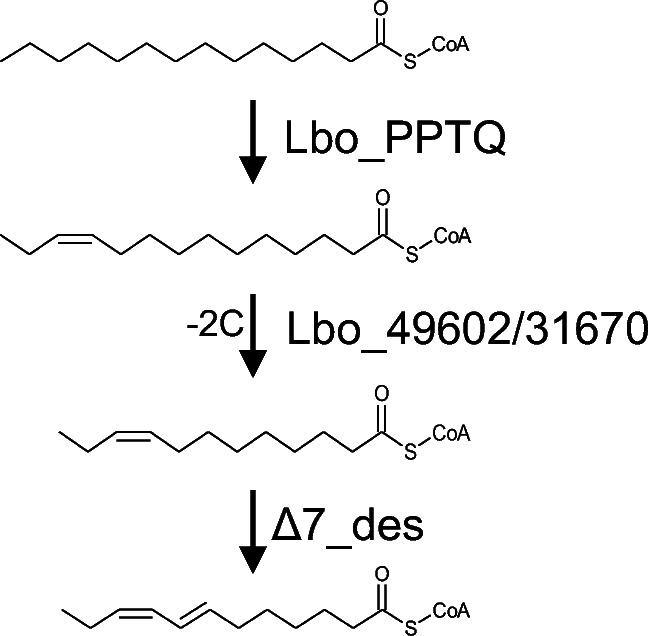
Proposed pathways for biosynthesis of the main pheromone component in *Lobesia botrana*

In the present study, we investigated the sex pheromone biosynthetic pathways in *L. botrana*. As shown in Fig. 9, our *in vivo* labeling experiment proved that the sex pheromone is biosynthesized from chain-shortening of 16:acid to 14:acid, followed by ∆11 desaturation to produce Z11–14:acid, which is further chain-shortened to Z9–12:acid. Subsequently, an unusual ∆7 desaturation occurs on Z9–12:acid to produce the precursor, E7,Z9–12:acid, which undergoes further reduction and acetylation to the corresponding alcohol and acetate.

Three geometrid moths, *Idaea aversata*, *I. straminata* and *I. biselata*, use different 7,9-dodecadienyl acetates as pheromone components, with (*Z,Z*)-9,11-tetradecadienyl acetate acting synergistically in field trapping of *I. aversata* (Ando et al. 1987; Biwer et al. 1975; Szőcs et al. 1987; Zhu et al. 1996). When D3–16:acid was applied to pheromone glands of *I. aversata*, label was incorporated into both Z9,Z11–14:OAc and Z7,Z9–12:OAc suggesting that, in this case, the dodecadienyl precursor is a chain-shortening product of the longer C_14_ homolog (Zhu et al. 1996). However, there is no evidence for a similar pathway in *L. botrana* because no (*E*,*Z*)-9,11-tetradecadienoic acid, neither native compound nor deuterium-labeled, was found in the pheromone gland after application of D_5_-Z11–14:acid. The incorporation of deuterium label from D_5_-Z11–14:acid into Z9–12:acid indicated chain-shortening of the monounsaturated tetradecenyl precursor to produce the dodecenyl intermediate (Fig. 2a). Trace-level label incorporation was found from D_3_–16:acid and D_3_–14:acid into E7,Z9–12:acid but not into Z9–12:acid, possibly because the amount of labeled monounsaturated intermediate was below the detection limit, or the ∆7 desaturase was highly active converting all the Z9–12:acid.

The subsequent functional assays of candidate genes in *∆ole1/elo1* yeast and Sf9 expression systems demonstrated that Lbo_PPTQ is a ∆11 desaturase working on 14:acid to produce Z11–14:acid, which is consistent with the results of the labeling experiments. In addition, our functional assays of all the other desaturase gene candidates confirmed that Lbo_KPSE, Lbo_NPVE, and Lbo_GATD are ∆9 desaturases, as suggested by phylogenetic analysis, and Lbo_LPGQ is a ∆11 desaturase forming predominantly Z11–18:acid and Z11–16:acid from 18:acid and 16:acid, respectively. Lbo_TPSQ is a ∆12 desaturase working on 14:acid, 16:acid and 18:acid. Although these showed desaturase activity in the functional assays, there was no evidence that these five ∆9, ∆11 and ∆12 desaturases are actually involved in pheromone biosynthesis in *L. botrana* based on the labeling experiments. Furthermore, no desaturase showed ∆7 desaturation activity on Z9–12:Me in our functional assays; E7,Z9–12:acid was not detected in any desaturase-transformed yeast when Z9–12:Me was added. Conjugated double bonds are formed in different ways in different moth species. For instance, many cloned and functionally characterized desaturases in moths are bi- or multi-functional ∆11 desaturases, preferably using 16:acid as substrate (Matoušková et al. 2007; Moto et al. 2004; Serra et al. 2006; Xia et al. 2019). In the codling moth, *Cydia pomonella*, (*E*,*E*)-8,10-dodecadienol (E8,E10–12:OH) is biosynthesized by a bifunctional E9 desaturase working on 12:acid (Löfstedt and Bengtsson 1988).

The aliphatic carbon chain length in moth pheromone compounds is adjusted by limited β-oxidation (Jurenka et al. 1994), which is considered to be performed by four enzymes, an acyl-CoA oxidase, an enoyl-CoA hydratase, a L-3-hydroxyacyl-CoA dehydrogenase, and a thiolase, as discussed in Ding and Löfstedt (2015), with several candidate genes suggested in *Agrotis segetum* in the same study. The first step of this β-oxidation is catalyzed by an acyl-CoA oxidase with different specificities (Osumi and Hashimoto 1978). As mentioned above, our labeling experiments demonstrated that, in the *L. botrana* pheromone gland, a β-oxidation enzyme was involved in producing the biosynthetic intermediate Z9–12:acid through chain shortening of Z11–14:acid. We found three full-length acyl-CoA oxidase (ACO) gene candidates from the transcriptome data, and heterologously expressed the two ACOs with the highest expression levels in the Sf9 system. The results showed that both Lbo_31670 and Lbo_49602 could chain-shorten Z11–14:acid to Z9–12:acid, but no shorter chain-length acids were found (Fig. 8).

Studies of pheromone biosynthetic pathways have demonstrated that chain-shortening is a crucial step in pheromone biosynthesis in many moth species (Bjostad and Roelofs 1983; Bjostad et al. 1987; Jurenka et al. 1994; Jurenka 1997; Xia et al. 2019; Wolf and Roelofs 1983; Wu et al. 1998). Differences in chain-shortening result in the production of different sex pheromone component ratios in two strains of the cabbage looper, *Trichoplusia ni*, i.e., the normal *T. ni* preferentially chain-shortened Z11–16:acid through two cycles of β-oxidation to Z7–12:acid, whereas mutant strain females had a reduced ability to chain-shorten (Jurenka et al. 1994). In the turnip moth, *Agrotis segetum*, successive β-oxidations starting from Z11–16:acid produced Z9–14:acid, Z7–12:acid, and Z5–10:acid. These three acids were then reduced and acetylated into the pheromone components Z9–14:OAc, Z7–12:OAc and Z5–10:OAc (Löfstedt et al. 1986). Differences in chain-shortening activity account for different ratios of these pheromone components in Swedish (12:59:29) and Zimbabwean (78:20:2) populations of *A. segetum* (Wu et al. 1998).

Identification of the genes encoding limited β-oxidation enzymes should help us understand the molecular control of chain shortening. The most important step here is the first, with the acyl-CoA oxidase catalyzing the formation of a double bond between the second and third carbon. However, this functionality had not previously been characterized in any moth species in the context of pheromone biosynthesis. This is the first study to report functional ACO genes involved in pheromone biosynthesis. We suggest that the subsequent three reactions are performed by the Sf9 cell machinery and most likely by a protein with three functions and less specific to substrate chain length (Hashimoto 1996).

To date, no acetyltransferase gene has been characterized in the context of moth sex pheromone biosynthesis. By homology, searching in *L. botrana*, we failed to find any novel candidate genes to test for this activity other than the ones from *A. segetum* that had previously been tested with negative results (Ding and Löfstedt 2015).

In conclusion, we reveal the biosynthetic pathway for the pheromone of the European grapevine moth, *L. botrana*, including evidence that an unusual ∆7 desaturation activity is involved. We found six functional desaturase genes, of which Lbo_PPTQ exhibits high ∆11 desaturase activity on tetradecanoic acid in sex pheromone biosynthesis in *L. botrana*. We also confirmed that Lbo_31670 and Lbo_49602 are key genes involved in chain shortening of Z11–14:acid for production of the biosynthetic intermediate Z9–12:acid, which sheds light on the enzymes involved in β-oxidation in pheromone biosynthesis in Lepidoptera. The molecular mechanism for introduction of the second double bond remains enigmatic. Possible explanations for our failure to identify and characterize the enzyme responsible for this reaction include the ∆7 desaturase not having the ‘normal’ motifs of other desaturases preventing its identification from the transcriptome or that our heterologous expression systems do not possess the necessary components to allow the successful functional expression of this desaturase.

## Supplementary Information

ESM 1 (DOC 147 kb)
