# Supplementary material for: Biosynthesis of the Sex Pheromone Component (E,Z)-7,9-Dodecadienyl Acetate in the European Grapevine Moth, Lobesia botrana, Involving ∆11 Desaturation and an Elusive ∆7 Desaturase
Source: J Chem Ecol. 2021 Mar 29;47(3):248–64. doi: 10.1007/s10886-021-01252-3 (PMC8019676; doi:10.1007/s10886-021-01252-3)
Supplement: Supplementary file 1 — (DOC 147 kb) [file 10886_2021_1252_MOESM1_ESM.doc]

**Biosynthesis of the Sex Pheromone (*E*,*Z*)-7,9-dodecadienyl acetate in the European Grapevine Moth *Lobesia botrana* involving ∆11 Desaturation and an Elusive ∆7** **Desaturase**

Bao-Jian Ding^1*^, Yi-Han Xia^1*^, Hong-Lei Wang^1^, Fredrik Andersson^2^, Erik Hedenström^2^, Jürgen Gross^3^, Christer Löfstedt^1^

*^1^ Department of Biology, Lund University, Sölvegatan 37, SE-223 62 Lund, Sweden.*

*^2^ Department of Chemical Engineering, Mid Sweden University, SE-851 70 Sundsvall, Sweden*

*^3^ Julius Kühn-Institut, Federal Research Centre for Cultivated Plants, Institute for Plant Protection in Fruit Crops and Viticulture, Dossenheim, Germany*

*Corresponding authors and shared first authorship

Bao-Jian Ding, [baojian.ding@biol.lu.se](mailto:baojian.ding@biol.lu.se)

Yi-Han Xia, [yihan.xia@biol.lu.se](mailto:yihan.xia@biol.lu.se)

**Description of the synthesis**

The deuterated unsaturated fatty acid **D_3_-Z9-12:acid** was prepared from methyl 9-hydroxynonanoate (Scheme 1).

Methyl 9-hydroxynonanoate was transformed to ester aldehyde **2**, in 71% yield, using Dess-Martin Periodinane as an oxidant.

By a Wittig reaction of the deuterium labelled reagent **2** [Reagent **2** was prepared in 92% yield, by reaction of equimolar amounts of triphenylphosphine and [3,3,3-^2^H_3_]-bromopropane in a sealed tube at 110 °C] and the aldehyde **1**,^2^ the deuterated unsaturated fatty methyl ester, **D_3_-Z9-12:Me**, was obtained in 33% yield, *E*:*Z* = 23:77. The stereochemical purity was increased by silica gel/AgNO_3_ chromatography to *E*:*Z* = 0.5:99.5.

Finally, the deuterated unsaturated fatty acid, **D_3_-Z9-12:acid** was obtained from **D_3_-Z9-12:Me** in 88% yield by basic hydrolysis.

1. T. Lucas and H. J. Schäfer, *Eur. J. Lipid Sci. Technol.* **2014**, 116, 52–62.
2. Y. M. A. W. Lamers et al., *Tetrahedron*, **2003**, 59, 9361–9369.

**Scheme 1.** (a) Dess-Martin Periodinane, CH_2_Cl_2_, 0 °C (71%); (b) **2**, KHMDS, THF, −78 °C (33%); (c) KOH, MeOH/H_2_O (88%); (d) [3,3,3-^2^H_3_]-bromopropane/PPh_3_ (sealed tube), 110 °C (92%).

**Experimental**

**Methyl 9-oxononanoate (1)**

Methyl 9-hydroxynonanoate (298 mg, 1.58 mmol), dissolved in CH_2_Cl_2_ (1 ml), was added to a suspension of Dess-Martin periodinane (741 mg, 1.71 mmol) in CH_2_Cl_2_ (10 ml) under an argon atmosphere at 0 °C. After stirring for 2 hours at 0 °C, stirring was continued for another 45 minutes at room temperature. n-Pentane (25 ml) was added to the reaction mixture and the formed precipitate was filtered off on a glass filter (P3). Evaporation of solvent at reduced pressure resulted in an oil with some residues of precipitate, which was immediately purified by flash chromatography (silica, EtOAc/cyclohexane), followed by bulb-to-bulb distillation (100 °C / 0.65 mbar). The title compound **1** was obtained as a clear colourless oil, 209 mg (98.3% purity according to GC, 71% yield).

^1^H NMR (500 MHz, CDCl_3_): 1.30-1.34 (6H, m,), 1.57-1.65 (4H, m), 2.30 (2H, t, *J* = 7.5 Hz), 2.41 (2H, dt, *J* = 1.8, 7.5 Hz), 3.66 (3H, s), 9.76 (1H, t, *J* = 1.8 Hz) ppm. ^13^C NMR (125.8 MHz, CDCl_3_): 21.98, 24.84, 28.90, 28.94, 28.99, 34.03, 43.87, 51.49, 174.23, 202.82 ppm. MS (EI) *m/z* (relative intensity): 186 (M^+^, not detected), 158 (16), 155 (31), 143 (48), 136 (11), 115 (14), 111 (48), 109 (12), 101 (12), 98 (15), 87 (78), 84 (13), 83 (47), 74 (100), 69 (28), 67 (20), 59 (29), 57 (15), 55 (59), 43 (30), 42 (11), 41 (33), 39 (12), 29 (16).

**Triphenyl([3,3,3-^2^H_3_]-propyl)phosphonium bromide (2)**

[3,3,3-^2^H_3_]-bromopropane (1.08 ml, 11.9 mmol) and triphenylphosphine (3.12 g, 11.9 mmol) were mixed together in a thick walled test tube, sealed with an aluminium septum cap, and heated in an oil bath (110 °C) for 23 hours. The resulting compact solid salt was grinded to a fine powder, washed with Et_2_O and dried (80 °C / 0.5 mbar) for 1 hour. The title compound **2** was obtained as a white powder, 4.24 g (92% yield).

^1^H NMR (500 MHz, CDCl_3_): 1.63-1.68 (2H, m,), 3.69-3.74 (2H, m), 7.66-7.70 (6H, m,), 7.75-7.83 (9H, m) ppm. ^13^C NMR (125.8 MHz, CDCl_3_): 16.38 (*J_P,C_* = 4.2 Hz), 24.49 (*J_P,C_* = 49.6 Hz), 118.40 (*J_P,C_* = 85.8 Hz), 130.58 (*J_P,C_* = 12.6 Hz), 133.74 (*J_P,C_* = 10.0 Hz), 135.09 (*J_P,C_* = 3.0 Hz) ppm.

**Methyl (*Z*)-9-[12,12,12-^2^H_3_] dodecenoate (D_3_-Z9-12:Me)**

KHMDS (1 M in THF, 5.17 ml, 5.17 mmol) was added to a suspension of phosphonium bromide **2** (2.01 g, 5.17 mmol) in THF (30 ml) at 0 °C under an argon atmosphere, and stirring was continued for another 2 hours. The red/orange ylide solution was cooled to ˗78 °C and aldehyde **1** (711 mg, 3.82 mmol) dissolved in THF (3 ml) was added. The temperature in the cooling bath was allowed to slowly increase in temperature and after 8.5 hours H_2_O (40 ml) was added. The organic layer was separated and the aqueous layer was extracted with Et_2_O (4 x 25 ml). The combined organic extracts were dried (MgSO_4_) and evaporation of solvent at reduced pressure resulted in a clear light yellow oil, which was purified by flash chromatography (silica, EtOAc/cyclohexane). The title compound **D_3_-Z9-12:Me** was isolated as a clear colourless oil, 268 mg (97.3% purity according to GC and *E*:*Z* = 23:77, 33% yield).

[13,13,14,14,14-

2

H

5

]-(Z)-11-Tetradecenoic acid

With further flash chromatography [10% AgNO_3_ on silica (10 g), 1-3% EtOAc/cyclohexane (80 ml of eluent for each percentage step), collecting ~3 ml/fraction. Elution order, *E*-isomer followed by *Z*-isomer], 118 mg of the title compound **D_3_-Z9-12:Me** could be obtained with high isomeric purity (98.8% purity according to GC and *E*:*Z* = 0.5:99.5).

GC retention times (HP-88 capillary column; 70 °C, 2 min, then programmed 10 °C/min up to 230 °C, 0 min): t_R_(*E*-isomer) 8.72 min and t_R_(*Z*-isomer) 9.01 min. ^1^H NMR (500 MHz, CDCl_3_): 1.30 (8H, br, -CH_2_(C*H_2_*)_4_CH_2_CH_2_CO_2_CH_3_), 1.62 (2H, quintet, *J* = 7.3 Hz, -CH_2_C*H_2_*CH_2_CO_2_CH_3_), 1.99-2.03 (4H, m, -CH_2_C*H_2_*CH=CHC*H_2_*CD_3_), 2.30 (2H, t, *J* = 7.6 Hz, -CH_2_C*H_2_*CO_2_CH_3_), 3.66 (3H, s, -CH_2_CO_2_C*H_3_*), 5.28-5.38 (2H, m, -CH_2_C*H=*C*H*CH_2_CD_3_, Irradiation of the allylic protons gave *J_cis_* = 10.8 Hz) ppm. ^13^C NMR (125.8 MHz, CDCl_3_): 20.40, 25.09, 27.19, 29.20, 29.27, 29.29, 29.82, 34.26, 51.61, 129.36, 131.74, 174.49 ppm. MS (EI) *m/z* (relative intensity): 215 (M^+^, 5), 184 (19), 183 (33), 141 (47), 123 (20), 110 (21), 98 (43), 97 (34), 96 (47), 87 (67), 84 (53), 83 (40), 74 (100), 72 (27), 69 (45), 59 (35), 58 (24), 55 (64), 43 (32), 42 (25), 41 (39), 31 (3), 29 (8).

**(*Z*)-9-[12,12,12-^2^H_3_] Dodecenoic acid (D_3_-Z9-12:acid)**

Deuterated methyl ester **D_3_-Z9-12:Me** (58 mg, 0.0.27 mmol) and 2.4 M KOH (10% H_2_O in MeOH, 1.5 ml) was stirred at room temperature for 19 hours. After evaporation of solvent at reduced pressure, H_2_O (3 ml) was added followed by extraction of the aqueous phase with EtOAc (3 ml). The aqueous phase was acidified with aqueous 2 M HCl and extracted with EtOAc (5 x 3 ml). The combined organic extracts were dried (MgSO_4_) and evaporation of solvent at reduced pressure resulted in a clear colourless oil, 48 mg (99.1% purity according to GC, 88% yield).

^1^H NMR (500 MHz, CDCl_3_): 1.31 (8H, br, -CH_2_(C*H_2_*)_4_CH_2_CH_2_CO_2_H), 1.63 (2H, quintet, *J* = 7.4 Hz, -CH_2_C*H_2_*CH_2_CO_2_H), 1.99-2.03 (4H, m, -CH_2_C*H_2_*CH=CHC*H_2_*CD_3_), 2.35 (2H, t, *J* = 7.5 Hz, -CH_2_C*H_2_*CO_2_H), 5.28-5.38 (2H, m, -CH_2_C*H=*C*H*CH_2_CD_3_, Irradiation of the allylic protons gave *J_cis_* = 10.8 Hz) ppm. ^13^C NMR (125.8 MHz, CDCl_3_): 20.40, 24.80, 27.18, 29.17, 29.27, 29.82, 34.17, 51.61, 129.34, 131.76, 180.21 ppm. 243 (M^+^, 6), 212 (26), 211 (35), 169 (38), 151 (8), 141 (13), 137 (14), 127 (25), 123 (21), 110 (30), 98 (49), 97 (48), 96 (57), 87 (73), 84 (54), 83 (42), 74 (100), 72 (32), 69 (59), 59 (35), 58 (26), 55 (76), 43 (38), 42 (26), 41 (44), 32 (2), 29 (9).
